# Risky Decisions and Their Consequences: Neural Processing by Boys with Antisocial Substance Disorder

**DOI:** 10.1371/journal.pone.0012835

**Published:** 2010-09-22

**Authors:** Thomas J. Crowley, Manish S. Dalwani, Susan K. Mikulich-Gilbertson, Yiping P. Du, Carl W. Lejuez, Kristen M. Raymond, Marie T. Banich

**Affiliations:** 1 Substance Dependence Division, Psychiatry Department, University of Colorado Denver, Denver, Colorado, United States of America; 2 Brain Imaging Center, University of Colorado Denver, Denver, Colorado, United States of America; 3 Psychology Department, University of Maryland, College Park, Maryland, United States of America; 4 Institute of Cognitive Science, Departments of Psychology and Neuroscience, University of Colorado Boulder, Boulder, Colorado, United States of America; University of California, San Francisco, United States of America

## Abstract

**Background:**

Adolescents with conduct and substance problems (“Antisocial Substance Disorder” (ASD)) repeatedly engage in risky antisocial and drug-using behaviors. We hypothesized that, during processing of risky decisions and resulting rewards and punishments, brain activation would differ between abstinent ASD boys and comparison boys.

**Methodology/Principal Findings:**

We compared 20 abstinent adolescent male patients in treatment for ASD with 20 community controls, examining rapid event-related blood-oxygen-level-dependent (BOLD) responses during functional magnetic resonance imaging. In 90 decision trials participants chose to make either a cautious response that earned one cent, or a risky response that would either gain 5 cents or lose 10 cents; odds of losing increased as the game progressed. We also examined those times when subjects experienced wins, or separately losses, from their risky choices. We contrasted decision trials against very similar comparison trials requiring no decisions, using whole-brain BOLD-response analyses of group differences, corrected for multiple comparisons. During decision-making ASD boys showed hypoactivation in numerous brain regions robustly activated by controls, including orbitofrontal and dorsolateral prefrontal cortices, anterior cingulate, basal ganglia, insula, amygdala, hippocampus, and cerebellum. While experiencing wins, ASD boys had significantly less activity than controls in anterior cingulate, temporal regions, and cerebellum, with more activity nowhere. During losses ASD boys had significantly more activity than controls in orbitofrontal cortex, dorsolateral prefrontal cortex, brain stem, and cerebellum, with less activity nowhere.

**Conclusions/Significance:**

Adolescent boys with ASD had extensive neural hypoactivity during risky decision-making, coupled with decreased activity during reward and increased activity during loss. These neural patterns may underlie the dangerous, excessive, sustained risk-taking of such boys. The findings suggest that the dysphoria, reward insensitivity, and suppressed neural activity observed among older addicted persons also characterize youths early in the development of substance use disorders.

## Introduction

Some 200,000 adolescent admissions annually occur in American substance-treatment programs [1]. Adolescent substance use disorders (SUD) are so strongly comorbid with antisocial conduct disorder (CD) [2]–[4] that the combination may be termed “antisocial substance disorder” (ASD). Both antecedent genetic influences [5]–[8] and toxic effects of drugs [9]–[12] may contribute to these behavioral problems, which often persist for decades [13]. ASD's great costs, both to those with the disorder and to society, make it important to understand this condition's etiology.

“Risky behaviors” are behaviors that may result unpredictably in rewarding and/or adverse outcomes. Adolescents generally tend to take more risks than adults, but in laboratories and in real life ASD youths, even when abstinent, take more risks than other adolescents [14], [15]. Indeed, ASD's symptoms of SUD and CD (*e.g.*, fire-setting, break-ins, and continued substance use despite problems [16]) epitomize extreme risky behaviors. Of note, ASD's risky behaviors are not necessarily “impulsive”, *i.e.*, done quickly without considering possible consequences. Indeed, they often require sustained preparation, such as “casing” a building before breaking in, or obtaining false identification to buy alcohol.

The excessive risky behaviors of ASD youths might result, first, from aberrant neural processing of behavior-motivating rewards; *e.g.*, among normal adolescents a risk-taking propensity does correlate with more reward-related activation of nucleus accumbens (NAc) [17] (also see [18]). Second, aberrant processing of behavior-inhibiting punishments could result in risky behaviors; *e.g.*, after punished responses in reversal learning, children with psychopathic traits show abnormally increased neural activation in ventromedial prefrontal cortex (vmPFC) and caudate [19] (also see [20]). Third, apart from initial processing of rewards or punishments, impaired integration of reward-punishment information in regions that decide on future behaviors could cause excessive risky behavior; *e.g.*, under risky conditions substance-dependent adults under-recruit specialized conflict-monitoring circuitry in posterior mesofrontal cortex [21]; also see [22], [23]. To address these three possibilities, we asked whether ASD youths under conditions of risk process decisions, rewards, or punishments differently from community-comparison youths.

Only a few studies have compared brain activation in ASD youths and controls. ASD youths did show greater activation in amygdala and regions of the default network while performing the Stroop task [24]. In a go/no-go task marijuana-using youths (without CD) had more activation frontally (and elsewhere) than controls [25]. Conversely, youths with familial risk for ASD had less frontal activation than controls during a motor inhibition task [26], perhaps like substance-involved adults who, when considering risky decisions, showed hypoactivity in brain regions processing potential losses and response conflicts [21].

Structural alterations of brain have been associated with the risk-taking of ASD youngsters, even among those merely vulnerable to ASD through family history. Youngsters with CD reportedly have reduced volume in insula and amygdala [27], and in temporal lobes, hippocampus, and vmPFC [28]. Compared with controls, alcohol-naïve sons of alcoholic men reportedly have widespread gray-matter volume reductions, the severity of which correlates with the severity of inattention, impulsivity, hyperactivity, and conduct problems [29]. Aggression and defiance negatively correlate with right ACC gray-matter volume among community boys not selected for ASD [30], while impulsivity negatively correlates with vmPFC volume [31].

Because ASD youths combine antisocial conduct problems with SUD, recent publications suggest partially conflicting possibilities for the neural underpinnings of their problems. First, like adults with antisocial or psychopathic traits (but substance-free) [32], ASD youths' repeated risk-taking might occur because they experience *increased dopaminergic response* to reward anticipation. Among antisocial adults impaired amygdala and vmPFC function also are thought [23] to reduce responses to punishment or loss. Increased response to reward and decreased response to punishment could cause excessive pursuit of exciting rewards with failure to inhibit behaviors that may be punished.

**Figure 1:**
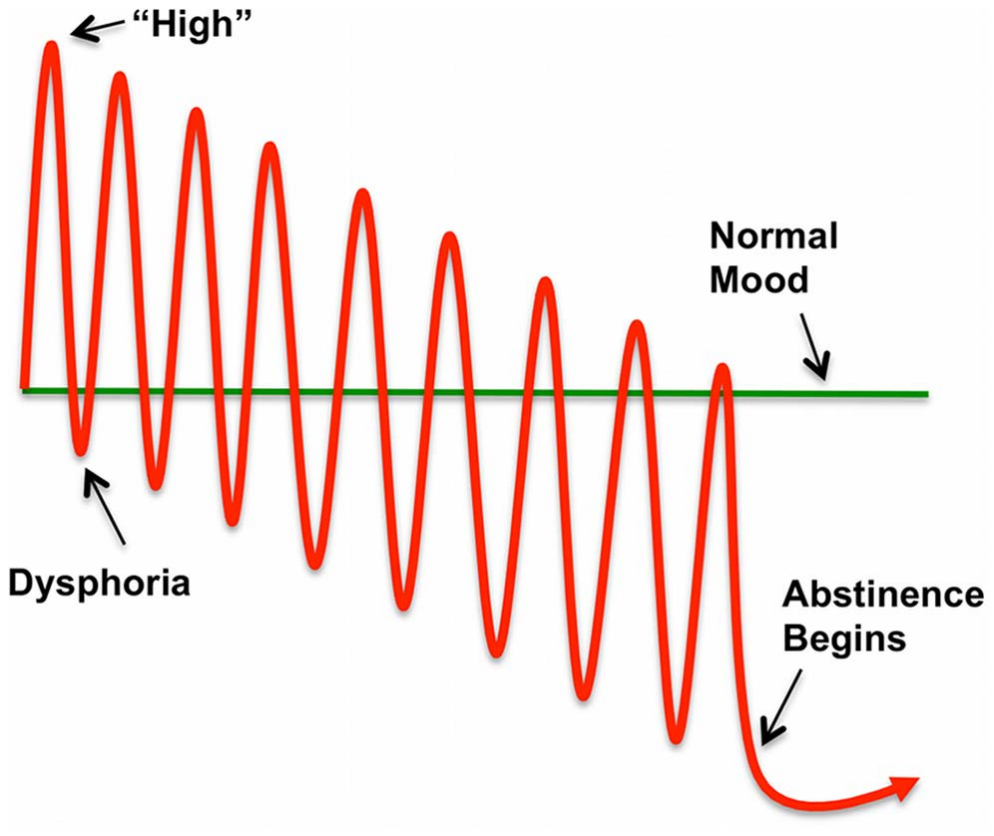
Schematic illustration of dysphoria induced by repeated intoxication-withdrawal cycles. Each intoxication leads to a subjective “high”, with enhanced response to reward due to reduced reward thresholds in medial forebrain bundle. Each acute withdrawal event results in subjective dysphoria with reduced response to reward due to elevated reward thresholds. Frequent cycle repetitions gradually suppress subjective “highs”, deepening dysphoria by further raising reward thresholds. Increasingly, the drug is used to escape dysphoria and achieve normal mood. During abstinence, mood recovers very slowly. (Based on Koob and Volkow [33]).

Alternatively, reviewing human and animal studies, Koob and Volkow [33] suggest that repeated intoxication-withdrawal cycles from addictive drugs are associated with *decreased dopaminergic response* to reward, due to increased stimulation thresholds in compromised reward circuits (see also [18]). These processes would produce “reward insensitivity”, reducing motivation for non-drug stimuli. Koob and Volkow [33] also indicate that chronic drug use disrupts frontal activity in ACC, OFC, and DLPFC, a disruption continuing well into protracted abstinence. Because those areas contribute to decision-making and behavioral inhibition, such disruption would facilitate recurring risk-taking and relapses. These authors further propose that repeated intoxication-withdrawal cycles activate a brain stress system mediated by corticotropin releasing factor (CRF) and other neurotransmitters [33]. They suggest that in human addicts hypodopaminergic reward insensitivity and stress activation present as subjective dysphoria, a “negative emotional state” that continues long into protracted abstinence (Fig. 1). Relapses at least briefly would relieve that dysphoria, negatively reinforcing further drug use (Fig. 1 and [34]).

With such conflicting suggestions in the literature, we could not make a directional hypothesis for this study. Thus, we simply hypothesized that, as adolescent boys repeatedly decide between doing a risky or a cautious behavior, and as they experience wins or losses from their risky choices, functional magnetic resonance imaging (fMRI) will show that youths with ASD have different brain activation patterns than community-control boys. Unlike some previous adolescent studies, our z-shim procedure [35] allowed good visualization of orbitofrontal regions that are important in processing reward and punishment [18]. Our results strongly supported our hypothesis.

## Methods

### Participants and Assessments

Ethics Statement: Written informed consent (adults) and assent (minors) was obtained from all research subjects. The Colorado Multiple Institutional Review Board approved all procedures.

Patients and controls were males, ages 14–18 years (inclusive) with IQ≥80, without known MRI contraindications (claustrophobia, orthodontic braces, color blindness, ferric metal in the body), and without history of unconsciousness >15 minutes, serious neurological illness, or neurosurgery. They and their parents spoke sufficient English for consenting. After explanation of procedures 18-year-old subjects provided written informed consent for participation; those <18 years old provided written assent and parents provided consent. Subjects were paid $50, won a mean of $6.25 more in the behavioral task, and earned $3 more if head movement was <2mm during the MRI.

Patients' inclusion criteria were: in treatment in our programs for youths (most referred by criminal-justice or social-service agencies and on probation); serious antisocial problems including DSM-IV [16] CD symptoms; DSM-IV [16] substance abuse or dependence on a non-nicotine substance; and multisubstance urine and saliva tests drug-free ≥7 days before assessment. Patients' exclusion criteria were: psychosis; current high risk of suicide, violence, or fire setting; or in treatment and abstinent ≥30 days (to minimize treatment effects on risk-taking). We obtained assent/consent on 28 patients, excluding 1 because of past embedded metal, 2 for not meeting substance diagnostic criteria, 4 for motion during imaging, and 1 for brain abnormalities noted during scanning. Twenty others completed all procedures.

To maximize similarity with patients we recruited *controls* in zip-code areas from which previous patients had come. One was referred by a previous control. All others were contacted by a telemarketing company that phoned, described the project, and invited families with possibly-qualifying children to accept a call from the researchers, who then met with the youth and a parent or guardian to explain the project, inviting written parental consent, and youth assent or consent, to participate. Regarding age, gender, English-language skills, and IQ, inclusion criteria were the same as patients'. Exclusion criteria were: court convictions (except minor traffic or curfew offenses); substance-related arrests, treatment, school-expulsions; obvious psychosis; physical illness; urine or breath tests containing non-prescribed substances a few days, or immediately, before scans; meeting criteria for DSM-IV CD in the last year; or non-tobacco substance dependence. As samples accumulated, we skewed control recruitment (*e.g.*, seeking older boys) to maintain patient-control comparability. Twenty-five control candidates provided assent/consent, but we excluded 1 for a substance-positive test, 2 for MRI-incompatible metal, 1 for motion during imaging, and 1 for signal loss from a large sinus; 20 others completed all procedures.

Psychosocial assessments were completed several days before fMRI's. Senior staff trained Bachelor-level interviewers and examined all records for accuracy. Typical interview time was 2 hrs for controls and 3 hrs for patients (who reported more symptoms). Assessments were: Child Behavior Checklist (CBCL) and Youth Self Report (YSR) [36], [37] for symptom severity of attention-deficit/hyperactivity disorder (ADHD), anxiety, and depression; Diagnostic Interview Schedule for Children (DISC-IV) [16], [37], [38] for CD symptoms and diagnoses; Composite International Diagnostic Interview-Substance Abuse Module (CIDI-SAM) [37], [39]–[41] for DSM-IV abuse or dependence for 11 substance categories; Peak Aggression Rating Scale [37]; Carroll Self-Rating Scale for depression severity [37], [42], [43]; Synergy Interview [37] for education, legal issues, and medical/psychological history; Modified Hollingshead-Redlich Social Class Rating [44]; Wechsler Abbreviated Scale of Intelligence (WASI) [45] Vocabulary and Matrix Reasoning for IQ estimates; Eysenck Junior Impulsiveness Scale [46]; and handedness preference [47].

Treating therapists tested patients' urine about weekly for substances. Researchers tested patients and controls with urine (AccuTest™) and saliva (AlcoScreen™) dipsticks about 1 week, and immediately, before scanning.

### Estimating Abstinence Duration

At treatment admission 14 patients, most referred from strictly controlled environments, produced an admission urine sample free of unprescribed drugs; 12 of those also denied any substance use in the previous 30 days and continued producing substance-free urine samples. For those 12 we estimated abstinence duration as: (30 days) + (number of days between admission and imaging). For all other patients abstinence duration was the length in days of a continuous series of during-treatment negative urine samples before imaging.

Of the four tobacco-experienced control subjects, one reported using tobacco regularly. All 20 patients reported smoking in the last 6 months, but 14 were now in a residential treatment program that vigorously suppressed smoking. Thus, we estimated that 6 non-residential patients and one control had used tobacco in the few days before imaging. No subjects smoked during the 1 hr pre-MRI training.

### Behavioral Tasks and Analyses

**Figure 2:**
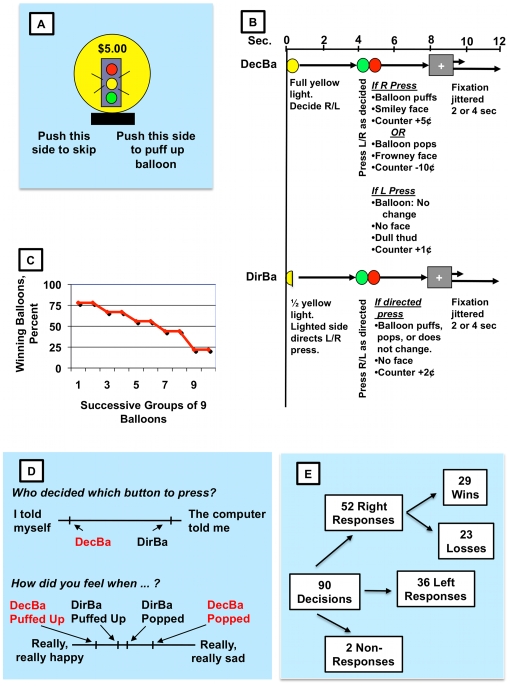
Colorado Balloon Game. **A.** Decision-Balloon screen, yellow light illuminated. Counter initially $5. **B.** Events during presentation of 90 paired trials, each Decision Balloon (DecBa) followed by a Directed Balloon (DirBa). Top: timing (seconds). Colored circles represent stoplight lights. DecBa begins (B, upper): yellow light illuminated 4 sec, subject decides to press left (L) or right (R) button. Green light (0.5 sec), subject executes response. Red light, consequence appears (3.5 sec). Risky right press consequence, either: (a) “smiley face”, expanding balloon, puffing sounds, counter adds 5 cents, or (b) “pop” sound, shrinking balloon, “frowney face”, counter loses 10 cents. Cautious left press consequence: +1 cent on counter, dull “thud” sound, unchanged balloon. Then, “jittered” fixation. DirBa's (B, lower) are identical to their paired DecBa's except: only half of initial yellow light illuminates, signaling (*i*) start of a DirBa and (*ii*) button to press during green light (*e.g.*, right illumination – press right) – the same button chosen during preceding paired DecBa. Green-light press on directed button: +2 cents on counter. Then balloon repeats the consequence (puff up, pop, or no change) of previous paired DecBa; subject was told that DirBa consequences would not affect earnings. Finally, jittered fixation screen. **C.** During DecBa, declining proportion of right presses programmed to win as game progresses. Mostly pressing left later in game saves earnings. **D.** Visual Analog Scales (VAS). After sessions subjects rated their opinions about the stated questions on 100mm lines. Marked positions represent all-subject means; groups did not differ significantly. Upper VAS: subjects' understanding of decision-making source for DecBa vs. DirBa. Lower VAS: Different emotional responses to puff-ups or pops of DecBa, vs. DirBa. **E.** Mean numbers, DecBa selections.

In a mock scanner subjects practiced our Colorado Balloon Game (CBG; Fig. 2), which is conceptually different from the Balloon Analogue Risk Task that we previously employed with similar patients [15]. We then conducted rapid event-related fMRI of neural processing (a) as subjects decided between doing a risky or a cautious behavior, and (b) as they experienced wins or losses from risky behaviors. “Decision Balloons” (DecBa) were test trials that forced a choice between doing a risky or a cautious behavior and then provided relatively large monetary wins or losses after risky behaviors. “Directed Balloons” (DirBa) were “baseline comparison” trials that required no decisions and provided only a small monetary reward for following a direction. DecBa and DirBa shared identical motor responses and almost identical visual and auditory stimuli (except for the initial full, vs. half, yellow light (Fig. 2B)), but only DecBa forced decisions and gave larger rewards or losses for risky decisions. Thus, we reasoned that subtracting baseline DirBa brain activation from DecBa activation should remove visual-, auditory-, and motor-related activation, while highlighting decision-related, and win-or-loss-related, activation.

### Imaging Neural Processing

Goggles and earphones delivered CBG's stimuli (Fig. 2A,B). The CBG presented 90 pairs of balloons, each pair including one DecBa (“You decide which button to press”) and one DirBa (“The computer will play the game. You don't need to decide anything”). Subjects responded with right and left index fingers on fiber-optic button response pads. Balloons within a pair usually were separated by balloons from other pairs (average 2.9, range 1–5, balloons, programmed with “optseq2” [48]. Each subject's session was divided into 3 runs, each presenting 30 identically-ordered DecBa-and-DirBa balloon pairs. Each DecBa or DirBa trial ended with a fixation screen (Fig. 2B), usually 2 sec, but in each of the subject's 3 runs four trials were “jittered” to 4 sec. Subjects received the amount on the counter at game's end; that amount could not fall below $3.00.

Across the 90 DecBa trials the reward schedule changed. To model real-life shifting of reward contingencies, risky right responses were very likely to be rewarded early, and punished later, in the game (Fig. 2C). Moreover, risky-response punishments (10 cents) were larger than rewards (5 cents) to further encourage gradual shifting from risky-right to cautious-left responding. Subjects only were advised, “Try to guess whether [the balloon] will pop from what the last few whole-yellow light balloons did. If the last few popped, maybe this one will pop. If the last few didn't pop, maybe this one won't pop.”

#### Imaging Decision-Making

During DecBa's 4-sec yellow light (Fig. 2B), subjects decided whether they would make a left or a right response when the green light came on. Since the yellow-light preceded responding, these 2 TRs reflected processing of decision, not response. DecBa was the test trial and DirBa was the “baseline comparison” trial; DirBa, unlike DecBa, required only compliance with a simple direction and no risky-vs.-cautious decision-making.

#### Imaging Reward-Punishment Processing

After the 4-sec yellow light (Fig. 2B) subjects responded during the 0.5-sec green light, and then during the 3.5-sec red light they observed the consequences (*risky right-response win*: smiley face, puff-up sound, balloon enlarges, counter increases 5 cents; *risky right-response loss*: frowney face, pop sound, balloon shrinks, counter decreases 10 cents; *cautious left response*: no face, dull thud sound, no change in balloon, counter increases 1 cent). Hence, combining the 2 TR's that spanned the green- and red-light periods (4 sec total; Fig. 2B) permitted us to assess the processing of reward or punishment (across-subject mean: 29 wins, and separately, 23 losses from 52 right presses (Fig. 2E)).

In the mock-scanner practice session subjects learned that during DirBa's red-light periods the counter increased 2 cents if the subject responded on the signaled side, regardless of subsequent audio-visual consequences (Fig. 2B); the latter were identical in each DecBa-DirBa pair. DirBa's always-predictable 2-cent reward for compliance was risk-free, certain, and considerably smaller than the 5-cent “win” reward, or the 10-cent “loss” punishment, that followed DecBa's risky choices. Accordingly, to assess win-or-loss related activation we subtracted DirBa activation from DecBa activation during the 4-sec green-and-red light period (Fig. 2B). In these analyses the first 0.5 sec included green-light motor responding, but in each pair of trials the DecBa and DirBa green-light stimuli and responses were identical (Fig. 2B), as were the red-light audio-visual stimuli. The only DecBa-DirBa difference was the meaning of those red-light stimuli (*DirBa*, 2-cent gain. *DecBa risky response*: 5 cent win or 10 cent loss; *DecBa cautious response*: 1 cent gain). Thus, subtracting DirBa activation from DecBa activation was designed to cancel out green-light-related activation, while highlighting activation associated with experiencing a win or a loss.

#### Other Data

We recorded occurrence of left or right responses and reaction times (from green-light onset to response), as well as the resulting consequences (*i.e.*, counter changes and the balloon's puff, pop, or no-change). Post-session debriefings asked about in-magnet experiences, game strategies, etc. On Visual Analogue Scales (VAS; Fig. 2D) subjects rated (a) the extent to which they or the computer made the left-right response decision for DecBa and for DirBa, and (b) their happy-sad reactions to balloon puff-ups or pops. T-tests and chi-square tests compared the groups on demographic and clinical variables, and on debriefing responses regarding DecBa and DirBa. Mixed models examined group and run differences (see below) in CBG's Total risky right presses, and last-session risky right presses.

### Image Acquisition

In a 3T General Electric MRI scanner, with stimuli synchronized to trigger pulses, subjects first observed a video during a 3D T1 anatomical scan (IR-SPGR, TR = 9 ms, TE = 1.9 ms, TI = 500 ms flip angle = 10°, matrix = 256×256, FOV = 220 mm^2^, 124 1.7 mm thick coronal slices; 9 min 12 sec).

Sessions then presented 90 paired DecBa-and-DirBa trials, divided into 3 runs. Each echo-planar (EPI) run (TR = 2000ms, TE = 26 ms, flip angle = 70°, FOV = 220 mm^2^, 64^2^ matrix, 36 slices, 4 mm thick, no gap, angled parallel to the planum sphenoidale) lasted 10 min, 23 sec, and had 30 paired DecBa-and-DirBa presentations. One-minute rest images (abstract nature drawing) separated the 3 runs.

Individual trials were discounted if the subject failed to respond behaviorally during the 0.5 sec green light. Data from individual trials with spike-like movement of the head >2 mm were replaced with dummy fixation data. The subject was excluded from analyses if 10 or more trials in a 30-trial run failed those criteria.

The 50-min session ended with T1 FLAIR images (T1-weighted spin-echo data set: 31 slices of part head, matrix = 256×192, NEX = 2, TE/TR/TI = 7.3ms/2000ms/860ms; imaging time = 4 min, 25 sec). Additionally, we acquired one IR-EPI (TR = 2000 ms, TE = 26 ms) volume (with excellent contrast between gray and white matter) to improve coregistration between EPIs and the IR-SPGR.

Our fast z-shimmed image acquisition was designed to reduce inferior frontal susceptibility artifact [35]. Compensation was applied only to a few slices covering the inferior frontal region to improve temporal resolution in a whole brain scan. Slice-acquisition order assured effective, constant repetition time in both the z-shim slices and other slices. Moreover, we applied z-shim compensation to 5 of the 31 slice locations in the OFC region. To optimize the amplitude of z-shim compensation gradient, G_c_, we ran on each subject a trial scan with 3 different G_c_ values (*i.e.*, 0.55, 0.70, and 0.85G_null_), where G_null_ is an amplitude that nulls the MRI signal in regions without susceptibility effect. We determined that a G_c_ of 0.70 G_null_ gave optimal signal recovery in the ventral-medial OFC. This G_c_ value produced robust OFC activation.

### Image Analysis

Data preprocessing included motion correction, coregistration to structural images, normalization to standard Montreal Neurological Institute (MNI) space, and smoothing. For within-subject fMRI analyses we fitted preprocessed data with the general linear model (GLM) of Statistical Parametric Mapping [49] software, filtering low frequency noise, correcting for temporal autocorrelation, and convolving with a single canonical HRF signal. A 128-s high pass filter removed signal drift and low-frequency fluctuation. The GLM model included these trial periods: decision, outcome (win or loss), and fixation. We generated single-subject contrast maps with SPM-2, analyzing brain-function differences in contrasts of interest (e.g. DecBa vs. DirBa) as fixed effects.

For between-subject whole-brain analyses, we compared groups' single-subject contrast maps generated by SPM2 using SPM5's random effects models. We used SPM5's ANCOVA to adjust all fMRI analyses for age and IQ (IQ mean: patients 97.1; controls 104.9, t (38) = 2.67; p = 0.011) before producing final statistical maps.

Agreeing that “research needs to move beyond the simple identification of single structures” [50], we conducted whole-brain, rather than region-of-interest, analyses, considering all structures exceeding cluster-defined thresholds. We expected to have greatest power in analyses that examined DecBa-DirBa differences in a single group of 20 subjects, considering all 90 trials together. For those analyses we controlled for false positive results with voxel-level family-wise error (FWE) correction (p_corr._<0.05).

In analyses expected to have less power (comparing 2 groups, and/or examining fewer trials), we used the cluster- level FWE correction (AFNI's AlphaSim program [51]), as used previously by us [24] and by others publishing in *PLoS One* or other excellent journals (*e.g.*, [52]–[57]). In comparison with voxel-level FWE, cluster-level FWE controls for false-positive results and achieves p_corr_<0.05 by simultaneously requiring a less significant difference in activation at each individual voxel (p_uncorr._<0.005), but also requiring a simulation-determined minimum number of contiguous activated voxels in each cluster. In other words, voxel-level FWE can identify an intensely activated, single-voxel “hotspot”; cluster-level FWE identifies multivoxel “warmspots” that, although less intensively activated, must be larger and so (like the voxel-level focus) would only occur by chance in whole brain at a multiple-comparisons-corrected probability of p_corr_<0.05. Considering our 6 mm full-width-half-maximum smoothing, 1000 Monte Carlo simulations estimated the overall significance level (probability of a false detection) for thresholding the 3D functional z-map image over the entire brain volume, regardless of activation within that map. These simulations indicated that requiring cluster size ≤97 voxels, and each voxel with an activation difference at p_(uncorr)_ = 0.005, provided a whole-brain family-wise corrected false positive rate p_(FWE-corr)_ = .05.

We examined potential confounds in our data with SPM's “glass-brain” images that show all beyond-threshold areas of activation. Those images were statistically adjusted with two continuous measures (ADHD severity (from CBCL) and depression severity (Carroll rating)). Three other potential confounds were categorical. For them we re-analyzed the data after excluding 3 left-handed subjects, and separately 7 current tobacco smokers, and separately after excluding subjects reporting prescribed medication use around the time of scanning (6 medicated patients (A used amphetamine-dextroamphetamine and risperidone; B, fexofenadine; C, fluoxetine, quetiapine; D, unidentified “ulcer drug”; E, methylphenidate; F, unidentified “asthma inhaler”) and 4 medicated controls (A and B, amphetamine-dextroamphetamine; C, albuterol; D, topiramate)).

Names for clusters' regions of maximum activation follow the NRW Research atlas [58]. Our “broad regions” include parts or all of Brodmann areas (BA) 10–14 and 47 in OFC [18]; portions of BA 6, 8, 9, and 46 in DLPFC [58]; inferior BA 8, and BA 44 and 45 in ventrolateral PFC (VLPFC) [59]; and portions of BA 6, 8, 9, and 10, and BA 24, 25, and 32 in medial prefrontal cortex (Med PFC) [60], [61]; BA 10 and 11, and the inferior and subgenual regions of ACC (BA 24 and 32) in vmPFC [23]. The atlas does not identify NAc; we considered it bounded by MNI coordinates x = (±) 4 to 15; y = 0 to 22; z = 2 to −10 [62]. Our procedures cannot resolve ventral tegmental area (VTA) and adjacent substantia nigra (SN) from surrounding structures, so we labeled the region within coordinates x = ±14, y = 14 to 28, z = −4 to −16 as “Midbrain (SN/VTA)” [63]; our “y” polarity is reversed from this reference. Rarely, SPM5 placed a cluster outside of gray matter (*e.g.*, in white matter), perhaps because of registration errors. If the cluster was ≤3mm from gray matter, we labeled it in the nearest gray matter; if it was >3mm, we do not report it.

Some of our data are expressed in standard SPM activation units. The mean activation of all brain voxels (white and gray matter) during the entire session is normalized at 100 percent, and mean activation in each region during DecBa, and separately during DirBa, is scaled proportionately in percentages. For example, if in some cortical region, patients' mean activation during DecBa is 180 percent of their mean activation in all brain voxels, and if their mean activation in that region during DirBa is 179 percent of mean activation in all brain voxels, then patients' mean DecBa-minus-DirBa activation difference in that region is 180-179 = 1 SPM activation unit.

## Results

### Demographics

All patients had been referred to our program for youths with serious antisocial and substance problems. Fourteen were in residential treatment, 4 in day-treatment, and 2 were outpatients. Patients and controls did not differ significantly in age or racial distribution (Table 1). However, patients' mean socioeconomic status score (equating to Social Class IV, lower middle class) was significantly lower than controls' score (III, upper middle class). As expected patients had significantly worse (Table 1) aggression and impulsiveness scores, conduct problems, number of CD symptoms, prevalence of CD, anxiety and depression (dysphoria) scores, attention problems, estimated mean IQ, number of SUD symptoms, and prevalence of SUD. Patients had many more legal problems.

**Table 1:** Subject characteristics.

	Patient	Control	Test	p-value
	N = 20	N = 20		
**Demographic and Psychiatric**				
Mean Age (SD)	16.5 (1.0)	16.5 (1.6)	t-test	NS
Caucasian (n)	12	15	chi-square	NS
Non-Caucasian (n)	8	5		
SES Score: Mean (SD)	47(18)	35(16)	t-test	p<.03
Social Class	III	IV		
Aggression Score: Mean (SD)	5.7 (3.2)	0.5 (1.1)	M-W U	p<.0005
Eysenck Impulsiveness Score: Mean (SD)	11.9 (6.0)	6.7 (4.5)	t-test	p<.005
Youth Self Report: CP Mean (SD)	69.0 (7.7)	53.8 (4.6)	t-test	p<.0005
CD Lifetime Symptom: Mean (SD)	6.8 (2.3)	0.5 (0.6)	M-W U	p<.0005
CD Lifetime Diagnosis (n^1^)	19	1	chi-square	p<.0005
CBCL, YSR^2^, Anx-Dep t-score: Mean (SD)	57.4 (8.6)	52.0 (7.3)	M-W U	p<0.007
CBCL, YSR^2^, Att-Prob Scale t-score: Mean (SD)	58.3 (8.2)	53.5 (4.5)	t-test	p = 0.029
Carroll Depression Rating Score: Mean (SD)	8.5 (6.7)	4.1 (3.8)	M-W U	p<.02
IQ full-scale t-score: Mean (SD)	97.1 (9.3)	104.9 (9.0)	t-test	p<.02
Sub Dep Symptoms, Across Drugs: Mean (SD)	12.4 (7.2)	0.2 (.67)	M-W U	p<.0005
**Substance Use Disorders** 3,4				
Tobacco Dependence	13	1	chi-square	p<.0005
Alcohol Abuse	8	0	Fisher Exact	p<.004
Alcohol Dependence	8	0	Fisher Exact	p<.004
Cannabis Abuse	7	0	Fisher Exact	p<.009
Cannabis Dependence	10	0	chi-square	p<.0005
Cocaine Abuse	2	0	Fisher Exact	NS
Cocaine Dependence	2	0	Fisher Exact	NS
Club Drugs Abuse	3	0	Fisher Exact	NS
Club Drug Dependence	4	0	Fisher Exact	NS
Hallucinogen Abuse	2	0	Fisher Exact	NS
Amphetamines Dependence	2	0	Fisher Exact	NS
Hallucinogen Dependence	1	0	Fisher Exact	NS
**Legal Problems** 5				
Lifetime Court Appearances: Mean (SD)	11 (11)	0		
Lifetime Admissions to Detention or Jail: Mean (SD)	3 (4)	0		
Days on Probation, Last 6 Months: Mean (SD)	139 (70)	0		

***Abbreviations***

Att-Prob, Attention Problems Scale. Anx-Dep, Anxious-Depressed Scale. CBCL, Child Behavior Checklist. CD, Conduct Disorder. CP, Conduct Problems t-score. M-W U, Mann-Whitney U test. SES, Socioeconomic Status. Sub Dep, Substance Dependence Symptoms. YSR, Youth Self-Report.

***Footnotes***

1 No controls met DSM-IV's past-year CD diagnostic criteria.

2 For one patient with no Child Behavior Checklist, Youth Self Report score was substituted.

3 For drugs not listed, no known cases.

4 Multiple disorders in some subjects, so numbers sum >20.

5 No statistical tests, due to lack of variance in control subjects.

Although symptom minimization is not uncommon in our patients' self-reports, 19 reported symptoms meeting criteria for DSM-IV conduct disorder (Table 1). Fourteen reported symptoms qualifying for DSM-IV substance dependence on at least one drug other than nicotine, and the other six reported symptoms qualifying for non-nicotine substance abuse without dependence. We conclude that these patients had ASD.

No subjects' urine or saliva contained alcohol or non-prescribed drugs just before scans. We estimated that patients were abstinent a mean of 38.6 (range 9–59) days before imaging. In the 30 days before imaging one control reported using alcohol on 2 days and another used cannabis on one day. We estimated that 6 patients and one control had used tobacco in the few days before imaging; they could use tobacco *ad libitum* before coming to the laboratory, but abstained for one hour before scans.

### Behavior

A post-fMRI VAS (Fig. 2D, upper line) showed that subjects understood that decisions were made by them in DecBa and not in DirBa (0mm = “I told myself”; 100mm = “the computer told me”; mean (±SD) scores (mm): DecBa, patients 6.5 (3.8), controls 12.1 (3.8); DirBa, patients 95.2 (5.0), controls 88.0 (5.0); DecBa or DirBa trial type F(1,38) = 333.8, p<0.0001; group NS). Another VAS (Fig 2D, lower line) showed that, as intended, the puff-ups or pops of DecBa produced stronger emotions than those of DirBa (0mm = “really, really happy”; 100mm = “really, really sad”. DecBa: DirBa x Puff: Pop interaction, F = 47.6 (df = 1,3); p<0.0001; patient-control main effect, NS). Hence, the data indicated that subjects clearly understood the different expectations of DecBa and DirBa trials, and that the rewards and punishments elicited the expected emotional responses.

Patients and controls did not differ in mean reaction times (Table 2). However, patients failed to respond within the 0.5 sec green-light limit on DecBa slightly but significantly more often than controls (patient mean 2.5 (±SD1.8) trials, controls 1.4 (±1.4), t(38) = 2.03, p = 0.049).

**Table 2:** Mean (SD) reaction times, msec.^a^

	Control	Patient	t-value	p
**DecBa** b	257 (30)	261 (26)	−0.47	NS
**DirBa** c	275 (28)	273 (31)	0.30	NS

***Footnotes***

a From green-light onset to response.

b All Decision Balloons with response during green-light period.

c All Directed Balloons with response during green-light period.

Considering all subjects together, the number of risky right presses decreased significantly across the three 30 trial runs. Mixed model analysis of risky right presses evaluated potential group, run, and group x run effects. Only the run effect was significant: F (1,40) = 49.0, p<0.0001. The estimated mean decrease was 7.3 (1.0) presses.

We thought that patients, compared to controls, might make more right presses overall, and especially on the last 30 DecBa trials. However, the groups did not differ in overall right responses (patients' mean (±SD) 52.2 (2.3); controls' 52.4 (1.9), t(38) = 0.07; NS), nor in right responses in the final 30-trial run (patients 15.1 (4.8); controls 15.0 (3.7), t (38) = −0.07; NS).

With no differences in right pressing, the groups did not differ significantly in wins (patients 28.3 (5.2); controls 29.9 (4.6); t (38) = −1.0; p = 0.32) or losses (patients 23.8 (6.0); controls 22.5 (4.8); t(38) = 0.76; p = 0.45). This similar win-loss experience helps in evaluating neural activation differences, since neither group experienced more frustration-inducing losses.

### Brain Activation During Decision-Making

In DecBa trials subjects decided about their next response during yellow-light periods, and in DirBa trials they were directed on how to respond during yellow-light periods. In many frontal and subcortical regions deciding recruited significantly more activation than following a direction. This was true for controls (Table 3; in this and each subsequent table, a footnote shows the contrast analyzed) and for patients, although patients activated many fewer voxels and regions (Table 4). The high t-values in these single-group, all-trial, FWE analyses reflect their considerable power. A formal two-group comparison of regions differently activated by controls and patients had less power than one-group analyses, and the stringent FWE procedure found no group differences. Therefore, we used a simulation procedure (see Methods) to determine a cluster-size threshold (≥97 contiguous voxels, each at p_uncorr_ = 0.005); such clusters were unlikely (p<0.05) to occur by chance in our whole-brain analyses.

**Table 3:** Controls' loci of activation during decision-making.A,B

Structure	Brodmann Area or Side^C^	Activated Voxels	Maximum Activation^D^			*t*
			*x*	*y*	*z*	
Sup & Mid Fr Gy	Mainly R 9, 10	293	36	54	10	19.1
Sup Fr Gy	Mainly R 10	65	26	52	32	11.5
Mid Fr Gy	L 10	16	−30	44	30	9.3
Mid Fr Gy	R 9	44	36	30	40	10.6
Med Fr Gy	R9	15	22	36	28	7.7
ACC^G^	Mainly R 24, 32	1776^G^	6	22	40	15.1
Med Fr Gy to Sup Fr Gy^G^	L, R 6, 8, 9		4	34	36	18.0
Sup Fr Gy (Pre-SMA)^G^	Mainly R 6		2	10	62	12.6
Inf Fr Gy^H^	R, L 45, 47	R 3260^H^; L 216^H^	36	24	−10	12.9
Insula^H^	R, L 13		40	18	2	11.7
N Ac^H^	R,L		12	14	−2	11.7
Caudate^H^	R, L		6	14	10	14.4
Putamen^H^	R, L		−14	6	−4	12.1
Midbrain (SN, VTA)^H^	R, L		10	−12	−14	11.0
Thalamus^H^	R, L	34	6	−12	14	13.7
Post Cing Gy	R	51	2	−26	30	9.4
Cereb Tonsil	L	39	−14	−52	−48	11.9
Cereb Ant Lobe^I^	R	43^I^	6	−60	−36	10.1
Uvula Vermis^I^	R		2	−64	−38	8.9
Cereb Tuber, Tonsil	L	194	−34	−64	−38	11.1
Clusters <10 voxels^E^	-	54	-	-	-	-
**Total Activated Voxels**	-	**6100**	-	-	-	-

***Abbreviations***

ACC, anterior cingulate cortex. Ant, anterior. Cereb, cerebellar. Cing, cingulate. Ctr, controls. DecBa, Decision Balloons. DirBa, Directed Balloons. Gy, gyrus. Inf, inferior. L, left. Med, medial. Mid, middle. N Ac, nucleus accumbens. Occ, occipital. Par, parietal. Post, posterior. Pt, patient. R, right. Sec, secondary. SMA, supplementary motor area. SN, substantia nigra. Sup, superior. Temp, temporal. Uncorr, uncorrected for multiple comparisons. VTA, ventral tegmental area.

***Footnotes***

A Procedure for determining significance: voxel-level family-wise error correction (p_corr_<0.05).

B Contrast examined: (DecBa)*_Ctr_* - (DirBa)*_Ctr_*.

C If bilateral, the largest maximum is shown.

D Montreal Neurological Institute coordinates, mm from anterior commissure.

E Combined volume of all clusters comprising <10 voxels.

G-J Regions bearing the same superscript comprise one activated cluster.

**Table 4:** Patients' loci of activation during decision-making.A,B

Structure	Brodmann Area or Side^C^	Cluster Size in Voxels	Maximum Activation^D^			*t*
			*x*	*y*	*z*	
Caudate, putamen, NAc	R	169	14	22	−10	11.1
Putamen, NAc	L	58	−16	18	−10	7.9
ACC^E^	Mainly R 24, 32	929^E^	6	28	34	15.3
Med Fr Gy^E^	Mainly R 6		0	16	50	8.9
Sup Fr Gy^E^	R 8		2	14	56	9.3
Inf Fr Gy	R, L 47	R 184; L 109	36	18	−8	12.6
Midbrain (SN, VTA)	R	18	4	−14	−10	8.5
Midbrain	L	10	−6	−12	−18	8.0
Midbrain	R	11	4	−26	−6	8.5
Clusters <10 voxels^F^	-	5	-	-	-	-
**Total Activated Voxels**	-	**1493**	-	-	-	-

***Abbreviations***: As in Table 3.

***Footnotes***

A Procedure for determining significance, as in Table 3.

B Contrast examined: (DecBa)_Pt_ - (DirBa)_Pt_.

C If bilateral, the largest maximum is shown.

D Montreal Neurological Institute coordinates, mm from anterior commissure.

E Regions bearing the same superscript comprise one activated cluster.

F Combined volume of all clusters comprising <10 voxels.

**Figure 3:**
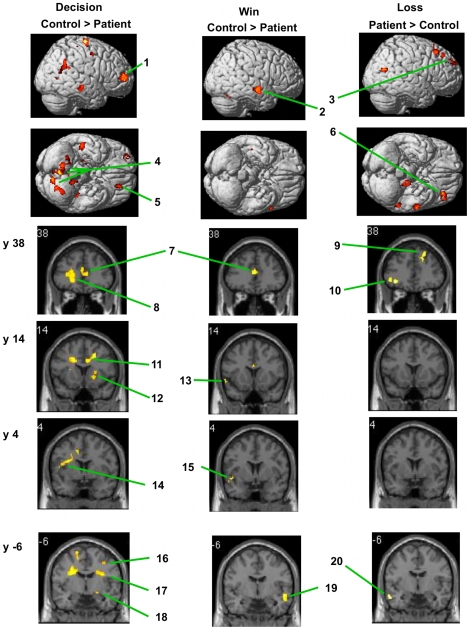
Selected regions more activated in one group (patients or controls) than in the other. Regions significantly more activated in controls than in patients: Left column, during decision-making (*cf.* Table 5 for contrast and details); middle column, while experiencing wins (*cf.* Table 8 for contrast and details). Regions significantly more activated in patients than in controls: right column, while experiencing losses (*cf.* Table 11 for contrast and details). Row 2: left is at bottom. Rows 3–6: left is at left. Values of “y”: for slices in that row, distance (mm) rostral (+) or caudal (−) from anterior commissure. Numbered regions: ***1***, middle frontal gyrus BA 10; ***2***, middle temporal gyrus BA 21; ***3***, medial frontal gyrus BA 9; ***4***, uvula (vermis) and pyramis; ***5***, medial frontal gyrus BA 10; ***6***, middle frontal gyrus BA 11, 47; ***7***, anterior cingulate BA 24, 32; ***8***, middle frontal gyrus BA 11; ***9***, superior frontal gyrus BA 8; ***10***, middle frontal gyrus BA 11, 47; ***11***, anterior cingulate BA 24; ***12***, putamen; ***13***, superior temporal gyrus, BA 38; ***14***, insula BA 13; ***15***, superior temporal gyrus BA 22; ***16***, middle frontal gyrus BA 6; ***17***, insula BA 13; ***18***, amygdala; ***19***, middle and inferior temporal gyri BA 21; ***20***, inferior temporal gyrus BA 21.

By that analysis, decision-making (compared to following a direction) activated a large set of regions significantly more among controls than among patients (Table 5; Fig. 3 (Decision)). Conversely, in the reverse contrast *no* brain regions activated more in patients than in controls. The discrepancy (controls>patients, 6233 voxels (Table 5); patients>controls, 0 voxels) strongly supports the conclusion that patients had less activation than controls during risky decision-making.

**Table 5:** Loci activating significantly more in controls than in patients during decision-making.A,B

Structure	Brodmann Area or Side^C^	Cluster Size in Voxels	Maximum Activation^D^			t
			*x*	*y*	*z*	
Sup Fr Gy^K^	R10	344^K^	21	54	6	3.8
Mid Fr Gy^K^	R10		32	50	6	4.1
Med Fr Gy^L^	L10	1724^L^	−20	42	−4	4.1
Mid Fr Gy^L^	L11		−24	38	−6	2.8
ACC^L^	L 24,32		−24	34	18	4.1
ACC^L^	R,L32		0	36	20	3.5
Insula^L^	L13		−46	−2	14	3.4
Claustrum^L^	L		−24	20	12	3.5
ACC^M^	R24, 32	444^M^	12	20	36	3.7
Med Fr Gy^M^	R9		20	36	24	2.9
Caudate^N^	R	137^N^	19	24	6	3.1
Putamen^N^	R		20	10	−2	3.2
Insula	R13	245	32	−6	22	2.8
Mid Fr Gy	R6	169	32	−2	48	3.7
Amygdala	R	118	22	−8	−12	3.2
Med Fr Gy^O^	L6	278^O^	−16	−10	58	4.0
Sup Fr Gy (Pre-SMA)^O^	L6		−18	−6	68	3.8
Pre-Central Gy^O^	L4		−16	−26	60	3.7
Med Fr Gy^P^	R6	369^P^	18	−16	60	3.5
Sup Fr Gy (Pre-SMA)^P^	R6		22	−14	71	4.2
Mid Temp Gy	R21	121	50	−24	−14	3.8
Hippocampus	L	157	−34	−32	−12	3.6
Post-Central Gy	L3	154	−60	−16	50	3.6
Post-Central Gy^Q^	L3	387^Q^	−32	−36	52	3.4
Inf Parietal Lobule^Q^	L40		−32	−52	58	3.2
Sup Temp Gy	L41	206	−42	−36	6	3.9
Precuneus^R^	L31	256^R^	−20	−46	30	4.0
Cing Gy^R^	L31		−20	−46	28	3.0
Supramarginal Gy	R40	175	60	−54	26	3.5
Lingual Gy	R19	126	30	−58	2	2.9
Ant Lobe	L	154	−8	−46	−32	3.4
Uvula Vermis^S^	R	327^S^	6	−62	−36	5.1
Culmen Vermis^S^	R		2	−62	−30	3.6
Post Lobe Cerebellar Tonsil^S^	R		16	−60	−48	4.2
Post Lobe Pyramis Vermis	L	342	−10	−76	−34	4.1
**Total Activated Voxels**	-	**6233**	-	-	-	-

***Abbreviations***: As in Table 3.

***Footnotes***

A Contrast examined: (DecBa-DirBa)_Ctr_ - (DecBa-DirBa)_Pt_.

B Procedure for determining significance: For voxel-wise uncorrected p<0.005 Monte Carlo simulations indicate that whole-brain clusterwise threshold p<0.05 (corrected for multiple comparisons) requires >96 clustered voxels.

C If bilateral, the largest maximum is shown.

D Montreal Neurological Institute corrdinates, mm from anterior commissure.

K-R Regions bearing the same superscript comprise one activated cluster.

**Figure 4:**
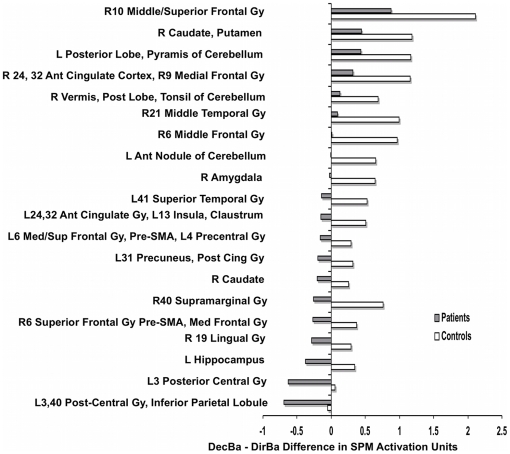
DecBa BOLD activity minus DirBa BOLD activity during yellow-light decision periods for patients and controls. Mean DecBa-minus-DirBa values are shown for patients, and separately for controls, in brain regions with significant control-patient differences (see Table 5). Negative deflections in some regions indicate that subjects' mean activity there was less during DecBa than during DirBa. Some regions extend across several anatomical structures. Some structures appear more than once because they contained more than one activated cluster. SPM activation units: see Methods. Abbreviations: Ant, anterior. Gy, gyrus. Med, medial. Pre-SMA, region immediately anterior to the supplementary motor area. Sup, superior.

To illustrate sources of group differences, Fig. 4 shows each group's mean DecBa-minus-DirBa activation difference (not adjusted for age or IQ) for each cluster in Table 3. In some regions both groups had more activation during decision-making (DecBa) than while following a direction (DirBa), but that difference was significantly greater among controls. Strikingly, however, in some regions patients' (but not controls') values were negative, indicating less activation during the decision-requiring DecBa than after the simple directions of DirBa.

To test whether patients' negative DecBa-minus-DirBa differences in Fig. 4 were due to abnormally high DirBa values in patients, we examined between-group differences in DirBa activation within the regions listed in Fig. 4. For each subject during DirBa, the mean activation within each region of interest was normalized (*i.e.*, expressed in “SPM activation units” (see Methods)). We then conducted between-group t-tests on those SPM activation values. In no brain regions did patients' DirBa values significantly exceed those of controls (data not shown). This suggests that, in comparison to controls, patients' negative DecBa-minus-DirBa values were due to reduced DecBa activation, rather than to enhanced DirBa activation.

### Brain Activation While Experiencing Wins or Losses

In the 90 DecBa trials subjects averaged 52 risky right responses, producing a mean of about 29 5-cent wins and 23 10-cent losses (Fig. 2E). We contrasted DecBa's 4-sec green-and-red light periods with those of DirBa, which paid 2 cents for each directed response. We analyzed wins, and separately losses, finding very distinct patterns in the patients and controls.

In the DecBa-minus-DirBa contrast, controls as a group significantly activated many structures, involving over 13,000 voxels, while patients activated fewer structures and about half as many voxels (Tables 6, 7). In a formal comparison seeking regions more activated by controls than patients, several regions and many voxels activated significantly (Table 8; Fig. 3 (Win)); the opposite contrast (patients>controls) found *no* regions activating significantly. These observations indicate that controls were more sensitive to wins than patients.

**Table 6:** Controls' loci of activation during Wins.A,B

Structure	Brodmann Area or Side^C^	Cluster Size in Voxels	Maximum Activation^D^			*t*
			*x*	*y*	*z*	
Mid Fr Gy	L 10	109	−36	56	12	5.2
Sup Fr Gy^H^	R 10	8618^H^	34	58	22	6.9
Mid Fr Gy^H^	R 11		24	54	−10	6.6
Sup Fr Gy^H^	R 9		24	52	38	7.0
Mid Fr Gy^H^	R 46		40	46	10	4.8
ACC^H^	R, L 32		0	44	12	4.6
ACC^H^	R, L 24,32		0	36	30	6.3
ACC^H^	R, L 33		0	20	24	4.9
Mid Fr Gy^H^	R 9		38	28	40	5.3
Sup Fr Gy^H^	R 6		6	23	59	4.6
Inf Fr Gy^H^	R, 47		40	22	−12	5.4
Mid Fr Gy^H^	R 8		40	20	52	5.1
Med Fr Gy^H^	Mainly R 8		2	18	52	5.0
Mid Fr Gy^H^	R6		38	4	62	4.9
Caudate^H^	R		18	10	16	5.5
Putamen^H^	R		28	2	6	6.5
Amygdala^H^	R		24	−12	12	3.4
Inf Fr Gy	L 47	151	−40	22	−12	5.0
Thalamus^I^	L	530	−2	10	12	3.5
Cing Gy^I^	R, L 23, 31		0	−34	34	3.7
Caudate^J^	L	1068^J^	−20	8	18	5.6
Inf Par Lobule^J^	L		−30	−30	42	3.9
Caudate Tail	R	146	36	−34	−4	5.0
Inf Par Lobule^K^	Mainly R 40	R 1653^K^; L 137^K^	48	−44	50	6.2
Supramarginal Gy^K^	R40		42	−48	38	6.1
Sup Par Lobule^K^	R 7		32	−74	52	4.1
Pyramis, Tonsil, Uvula	L	504	−40	−68	−44	7.1
Declive, Culmen	R	115	38	−66	−28	3.7
Cuneus	R19	144	6	−82	36	4.0
Inf/Mid Occ Gy	R 18	355	34	−92	−8	4.4
**Total Activated Voxels**	-	**13530**	-	-	-	-

***Abbreviations*** *:* As in Table 3.

***Footnotes***

A Procedure for determining significance: as in Table 5.

B Contrast Examined: (DecBa Rt-Resp Win Trials)*_Ctr_* - (Paired DirBa Rt-Resp Trials)*_Ctr_*.

C If bilateral, the largest maximum is shown.

D Montreal Neurological Institute coordinates, mm from anterior commissure.

H-K Regions bearing the same superscript comprise one activated cluster.

**Table 7:** Patients' loci of activation during Wins.A,B

Structure	Brodmann Area or Side^C^	Cluster Size in Voxels	Maximum Activation^D^			*t*
			*x*	*y*	*z*	
Mid Fr Gy^L^	R 10	2649^L^	22	52	−6	4.7
Sup Fr Gy^L^	R 10		38	52	18	5.0
Mid Fr Gy^L^	R 11		22	48	−14	4.3
Sup Fr Gy^L^	R9		44	36	38	4.1
Mid Fr Gy^L^	R8		36	22	48	4.8
Caudate^L^	R		18	8	20	3.4
Inf Fr Gy^M^	R 47	790^M^	42	20	−14	7.6
Amygdala^M^	R		26	2	−17	3.4
Putamen^M^	R		28	0	−2	4.9
Inf Fr Gy	L 47	345	−38	20	−8	5.1
Putamen	L	117	−30	−12	0	3.9
Mid Fr Gy	R 6	173	28	4	64	4.0
Precentral Gy	L 6	776	−42	−2	32	3.7
Thalamus	L, R	374	4	−4	10	3.2
Sup Parietal Lob^O^	R, L 7	R 788^O^; L 359^O^	42	−60	58	6.1
Precuneus^O^	R, L		32	−74	52	3.0
Uvula	R, L	139	−6	−68	−44	4.2
Cereb, Post Lobe, Declive	R, L	106	−2	−78	−16	4.2
**Total Activated Voxels**	-	**6616**	-	-	-	-

***Abbreviations*** *:* As in Table 3.

***Footnotes***

A Procedure for determining significance: as in Table 5.

B Contrast Examined: (DecBa Rt-Resp Win Trials)*_Pt_* - (Paired DirBa Rt-Resp Trials)*_Pt_*.

C If bilateral, the largest maximum is shown.

D Montreal Neurological Institute coordinates, mm from anterior commissure.

L-O Regions bearing the same superscript comprise one activated cluster.

**Table 8:** Loci activating significantly more in controls than in patients during Wins.A,B

Structure	Brodmann Area or Side^C^	Cluster Size in Voxels	Maximum Activation^D^			*t*
			*x*	*y*	*z*	
ACC	R, L, 24, 32	215	2	34	20	4.01
Sup Temp Gy	L 22, 38	103	−48	8	−6	4.66
Sup Temp Gy^P^	R 22	168^P^	54	−6	−8	3.78
Mid Temp Gy^P^	R 21		54	−8	−18	2.85
Inf Temp Gy^P^	R 21		58	−10	−18	2.84
Precuneus	R 31	105	20	−72	22	3.67
Fusiform Gy^Q^	R 19	286^Q^	22	−66	−14	4.23
Declive^Q^	R		30	−62	−22	3.83
Declive	L	102	−28	−64	−26	3.58
**Total Activated Voxels**	-	**979**	-	-	-	-

***Abbreviations*** *:* As in Table 3.

***Footnotes***

A Procedure for determining significance: as in Table 5.

B Contrast Examined: [(DecBa Rt-Resp Win Trials)*_Ctr_* - (Paired DirBa Rt-Resp Trials)*_Ctr_*] - [(DecBa Rt-Resp Win Trials)*_Pt_* - (DirBa Rt-Resp Trials)*_Pt_*].

C If bilateral, the largest maximum is shown.

D Montreal Neurological Institute coordinates, mm from anterior commissure.

P-Q Regions bearing the same superscript comprise one activated cluster.

In controls losses (Table 9) activated fewer structures and voxels than wins (Table 6). Moreover, unlike wins, losses actually activated slightly fewer voxels in controls (Table 9) than in patients (Table 10). Indeed, in formal comparisons of the two groups we found *no* voxels more activated in controls than in patients, while the patient>control contrast found many activated structures and voxels (Table 11; Fig. 3 (Loss)); the largest cluster was in prefrontal cortex. These findings indicate that patients were more sensitive to losses than controls.

**Table 9:** Controls' loci of activation during Losses.A,B

Structure	Brodmann Area or Side^C^	Cluster Size in Voxels	Maximum Activation^D^			*t*
			*x*	*y*	*z*	
ACC	R, L, 24,32	1716	4	38	20	8.1
Sup Fr Gy	R, L 6	384	10	4	72	5.7
Inf Fr Gy^M^	R 47	504^M^	42	14	−10	5.9
Sup Temp Gy^M^	R 38		36	14	−26	3.3
Insula^M^	R 13		40	12	−4	5.5
Inf Fr Gy	L 38, 47	267	−36	20	120	6.1
Midbrain (SN/VTA)^N^	R, L	412^N^	0	−30	−8	6.0
Culmen, Ant Lobe^N^	R, L		−6	−38	−14	3.3
Tuber	L	104	−34	−60	−38	4.3
**Total Activated Voxels**	-	**3387**	-	-	-	-

***Abbreviations*** *:* As in Table 3.

***Footnotes***

A Procedure for determining significance: as in Table 5.

B Contrast Examined: (DecBa Rt-Resp Loss Trials)*_Ct_*_r_ - (Paired DirBa Rt-Resp Trials)*_Ctr_*.

C If bilateral, the largest maximum is shown.

D Montreal Neurological Institute coordinates, mm from anterior commissure.

M-N Regions bearing the same superscript comprise one activated cluster.

**Table 10:** Patients' loci of activation during Losses.A,B

Structure	Brodmann Area or Side^C^	Cluster Size in Voxels	Maximum Activation^D^			*t*
			*x*	*y*	*z*	
ACC^O^	R, L, 24, 32	2114^O^	0	26	36	8.2
Sup Fr Gy^O^	R 9		22	34	38	3.2
Med Fr Gy^0^	R, L 9		2	34	36	5.2
Inf Fr Gy^p^	L 47	301^p^	−42	18	−14	5.7
Sup Temp Gy^p^	L 38		−38	18	−24	3.3
Sup Temp Gy^Q^	R 38	613^Q^	32	12	−34	5.6
Inf Fr Gy^Q^	R 47		36	16	−18	5.1
Sup Fr Gy	R 6	257	24	6	58	4.5
Midbrain (SN/VTA)^R^	R, L	290^R^	2	−34	−8	4.3
Ant Lobe Culmen^R^	R, L		4	−46	−2	5.3
**Total Activated Voxels**	-	**3575**	-	-	-	-

***Abbreviations*** *:* As in Table 3.

***Footnotes***

A Procedure for determining significance: as in Table 5.

B Contrast Examined: (DecBa Rt-Resp Loss Trials)*_Pt_* - (DirBa Rt-Resp Loss Trials)*_Pt_*.

C If bilateral, the largest maximum is shown.

D Montreal Neurological Institute coordinates, mm from anterior commissure.

O-R Regions bearing the same superscript comprise one activated cluster.

**Table 11:** Loci activating significantly more in patients than in controls during Losses.A,B

Structure	Brodmann Area or Side^C^	Cluster Size in Voxels	Maximum Activation^D^			*t*
			*x*	*y*	*z*	
Sup Fr Gy^s^	L 10	430^s^	−8	62	30	3.5
Sup Fr Gy^s^	R 9		8	50	34	3.2
Med Fr Gy^S^	R 9		12	46	30	3.7
Sup Fr Gy^S^	R 8		22	38	44	3.2
Mid Fr Gy^S^	R 8		18	24	46	3.7
Mid Fr Gy	L 11, 47	108	−34	38	−8	4.0
Mid to Inf Temp Gy	L 21	114	−58	−6	−22	4.2
Brainstem, Pons^T^	L	115^T^	−16	−26	−30	4.2
Culmen^T^	L		−20	−34	−26	3.3
Paracentral Lobule^U^	R 31	111^U^	2	−32	46	3.5
Cing Gy^U^	R 31		6	−34	40	3.9
Mid Temp Gy	L 21	128	−58	−40	−6	3.2
Mid Temp Gy	R 39	120	46	−64	20	2.8
Precuneus	L 7	107	−4	−62	44	3.8
**Total Activated Voxels**	-	**1233**	-	-	-	-

***Abbreviations*** *:* As in Table 3.

***Footnotes***

A Procedure for determining significance: as in Table 5.

B Contrast examined: [(DecBa Rt-Resp Loss Trials)*_Pt_* - (DirBa Rt-Resp Loss Trials)*_Pt_*] - [(DecBa Rt-Resp Loss Trials)*_Ctr_* - (DirBa Rt-Resp Loss Trials)*_Ctr_*].

C If bilateral, the largest maximum is shown.

D Montreal Neurological Institute coordinates, mm from anterior commissure.

S-U Regions bearing the same superscript comprise one activated cluster.

### Possible Confounds

**Figure 5:**
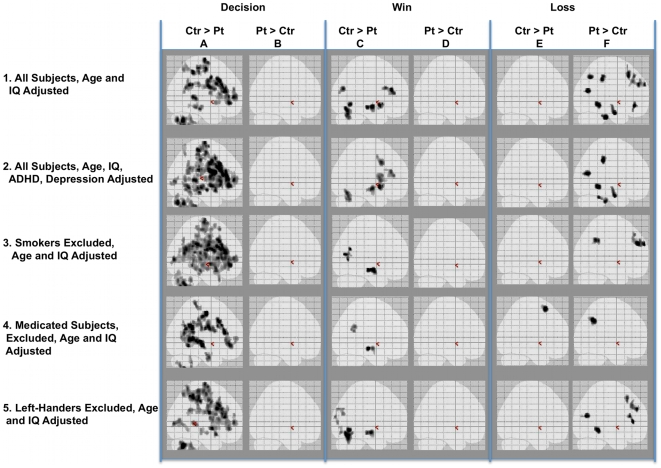
Influences of ADHD, depression, tobacco smoking, prescription medications, or handedness on control-patient group differences. Right sagittal view. Explanation in Methods and Results.

Compared with controls, patients' neural function was reduced during decision-making and wins, and enhanced during losses, and we sought confounds that might explain these differences. “Glass brains” (Fig. 5), 2-dimensional shadowgrams of all activated areas, obscure details but visually summarize important large-scale patterns. The shadowgram in Fig. 5, Cell 1A (Row 1, Column A), presents the data of Table 5 (Decision period, all trials, control>patient activation regions), showing numerous activated regions. Cell 1B shows the reverse (patient>control) contrast, and no regions activate. The stark differences between Cells 1A and 1B persisted when we simultaneously adjusted brain activity for ratings of depression and ADHD severity (Fig. 5, Cells 2A, 2B); or after we excluded 6 patients and 1 control thought to be current, regular cigarette smokers (Cells 3A, 3B); or after exclusion of 6 patients and 4 controls using prescription medications (Cells 4A, 4B); or after exclusion of 3 left-handed subjects (Cells 5A, 5B).

Similarly, Fig. 5, Cell 1C, shows regions in which controls' activation exceeded patients' during wins (from Table 8), and Cell 1D shows the reverse (patient>control) contrast. Again, the complete absence of patient>control activity in Cell 1D carries down after adjustment for ADHD and depression (Cell 2D), or after we excluded smokers (Cell 3D), or medicated subjects (Cell 4D), or left-handers (Cell 5D).

Finally, the shadowgram of Fig. 5, Cell 1E, shows that during losses controls' activation exceeded patients' in no regions, whereas many areas were activated in the patient>control contrast (Cell 1F). These differences persisted when we adjusted for ADHD and depression (Cells 2E, 2F), or when we excluded smokers (Cells 3D, 3F) or left-handers (Cells 5D, 5F). The pattern was broken only after we excluded 10 medicated subjects; then, among the remaining 10 there was greater activation in right superior frontal gyrus (BA6) among controls, compared with patients (Cell 4E). However, that single finding, relying on only 10 subjects and an average of 23 DecBa-DirBa trials, does not negate our conclusion: it appears unlikely that these potential confounds explain the large patient-control neural-activation differences that we report.

## Discussion

In this fMRI study we investigated the hypothesis that, while deciding between doing a risky or a cautious behavior, or while experiencing wins or losses from risky choices, youths with ASD would have different brain activation patterns than community-control boys. Important design features included an adolescent sample with very serious antisocial and substance problems, a z-shim procedure to enhance orbitofrontal imaging, and a novel task that compared risk-taking trials with almost-identical risk-free trials. We discuss three main findings.

### Patients' Neural Hypoactivity During Decision-Making

While deciding between doing a risky or a cautious behavior, patients' brains showed extensive neural hypoactivity. Koob and Volkow [33] predicted that during protracted abstinence addicts would show “disrupted activity of frontal regions, including dorsolateral prefrontal regions, cingulate gyrus, and orbitofrontal cortex”, a disruption “hypothesized to underlie their impaired inhibitory control and impulsivity … [contributing] to relapse”.

Per those predictions, our patients activated about 4-fold fewer voxels than controls during decision-making (Tables 3 and 4). Group comparisons with a cluster-based threshold found almost 6000 voxels more activated in controls than patients (Table 5), and none more activated in patients than in controls. As in the Koob-Volkow predictions, while making decisions patients activated right DLPFC and bilateral ACC, as well as left OFC (medial frontal gyrus, BA 10) significantly less than controls (Table 5). DLPFC generates “higher order cognitive processes that regulate the selection among multiple competing responses and stimuli” [59]. It is part of a complex “executive”, “control” [18], or “STOP” [64] system that, among other things, inhibits behavior. ACC monitors rewards and punishments, signaling DLPFC to adjust behavior to maximize future rewards [60]. Dysfunction in those regions could contribute to disinhibited antisocial and drug-using behaviors.

However, the activation difference between our groups extended well beyond the frontal regions predicted by Koob and Volkow [33]. We next suggest that, along with DLPFC, OFC, and ACC, patients' decision-related hypoactivity in other regions (Table 5) may contribute to their faulty real-life decision-making.

While making decisions patients had hypoactivity in insula, which usually co-activates with ACC. Via widespread connections [65] insula integrates current internal feelings with past memories to guide goal-directed behavior [66]. Insula may assess risks before behavior selections, evaluating possible losses or punishments and signaling the probability of aversive outcomes [66]–[71]; it activates in anticipation of risk in betting games [72]. Insula's anatomic volume is reduced among children with conduct disorder [27].

As behaviors are learned, becoming habitual, their control gradually shifts to caudate and putamen, “the core neurobiological substrate of both goal-directed and habitual control of instrumental responding” [73]. Caudate frequently activates with risk decisions [74]. Caudate and putamen were hypoactive among patients during decision-making.

Amygdala, together with OFC and NAc, processes cues that predict positive or negative outcomes in goal-directed behavior, guiding decisions with cue-induced positive and negative emotional memories [75]; amygdala damage impairs the use of emotional memories as animals choose among behaviors [76]. Dysfunction in amygdala and associated temporal cortical regions are considered central to psychopathy [23]. Meanwhile, hippocampus processes memories of environmental (“contextual”) cues [75] to guide behavior. Alcoholic parents' at-risk offspring have reduced amygdala and hippocampus volumes even before significant alcohol use [29], and children with conduct disorder also have reduced amygdala volumes [27], [28]. Amygdala, hippocampus, and temporal cortical structures all were hypoactive as patients made decisions (Table 5).

Also hypoactive was patients' pre-supplementary-motor area (pre-SMA), which projects to ACC, insula, lateral prefrontal regions, caudate, and putamen [77]. Using that network pre-SMA integrates choice or volition with organized movement. Many subjects reported shifting response strategies during CBG, and pre-SMA activates as subjects switch between different stimulus-response rules. Pre-SMA also activates more before self-selected movements (such as DecBa responses) than before externally-commanded ones (like DirBa responses).

Post-central gyrus (BA 3), middle occipital gyrus (BA19), and inferior parietal lobule (BA40) all activate during risk-taking tasks [74]. Deficits in inferior parietal lobule may contribute to antisocial behavior [50]. All were hypoactive as our patients made decisions.

BA31 (including cingulate gyrus and adjacent precuneus) is part of a broad posteromedial cortex [78], [79] with very wide connections. BA31 therefore has “the means to influence, and be influenced by, an extensive network of cortical structures involved in processing highly integrated and associative information”; it appears to influence “notion of self” [78], including “first person perspective-taking and experience of agency” [79], functions relevant in behavior-inhibition disorders. BA31 was hypoactive during patients' decision-making.

Interconnected with PFC, cerebellum (especially midline vermis) has an executive role in planning, problem solving, working memory, and mental flexibility [80]; cerebellar damage may produce disinhibition, inappropriate comments, and impulsivity. Cerebellum was hypoactive as patients made decisions.

An unexpected contribution to patients' relative neural hypoactivity during decision-making was that in numerous areas DecBa generated less BOLD activity than DirBa, resulting in a negative DecBa-minus-DirBa difference (Fig. 4). DecBa required complex choices. DirBa, a low-demand comparison, required only simple motor responses to simple directions. Nevertheless, in several key brain regions patients' neural activity was stronger during DirBa than during DecBa (Fig. 4). This apparently was not due to elevated DirBa activation in patients; DirBa activation, as a percentage of mean activation in all brain voxels, was similar for patients and controls. So among youths with ASD it appears that (in comparison to simple directions) risky-choice opportunities actually *reduced* BOLD activity in some regions, perhaps contributing to these youths' frequent real-life risk-taking. Whether risk-free choices similarly would reduce neural activity among patients remains a question for future research.

### Patients' Dysphoria, Reward Insensitivity, and Loss Hypersensitivity

Compared with controls, patients registered greater dysphoria on both a depression scale and an anxious-depressed scale (Table 1), and their BOLD responses showed both reduced sensitivity to reward and heightened sensitivity to punishment. Koob and Volkow [33], reviewing studies of drug self-administration by animals, find consistent evidence for both hypodopaminergic reward insensitivity and CRF-related activation of a brain stress system, and they propose that in human addicts these processes manifest as subjective dysphoria.

#### Experiencing Wins

While experiencing wins, controls activated numerous structures, including a single massive, mainly right-sided cluster involving DLPFC, VLPFC, OFC, ACC, dorsal striatum, and amygdala (Table 6). During wins, although patients also activated many of these structures, they activated only about half as many voxels (Table 7). Patients activated no regions more than controls. Meanwhile, controls activated ACC significantly more than patients (Table 8); ACC monitors reinforcements unexpectedly delivered or omitted, signaling lateral PFC to adjust behavior to maximize rewards [60]. Disruption of that signaling may relate to patients' real-life repetition, despite frequent punishment, of antisocial and drug-using behaviors. Controls also exceeded patients in activating temporal and parietal association regions, precuneus, fusiform gyrus, and cerebellum (Table 8), regions known to process reward-related stimuli [23], [81]–[83].

These “win” findings further support the Koob-Volkow [33] arguments. Patients showed the predicted dysphoria (Table 1; *cf.*, Fig. 1) and the predicted reduction in ACC activity (Table 8). Patients' widespread brain hypoactivity during win experiences reflected “reward insensitivity”.

In familiar tasks the dopaminergic NAc and midbrain VTA/SN regions typically activate with stimuli that predict a reward, rather than upon reward delivery [84]. Thus, as expected, our patients and controls did not activate these regions upon reward delivery (Tables 6, 7). Instead, in both groups the regions activated in *anticipation* of reward, during the yellow-light decision period (Tables 3, 4). But strikingly, those BOLD responses in the two groups were not significantly different (Table 5). Apparently, they similarly processed reward anticipation in NAc and VTA/SN, and that may appear to challenge suggestions of patients' reward insensitivity. However, VTA/SN also project to caudate and putamen, regions recently recognized as important in reward-based decision making [85], and those regions did activate significantly less in patients than in controls during the yellow-light period of decision-making and reward anticipation (Table 5); patients were relatively insensitive to reward anticipation there.

Why then did the NAc BOLD response of the two groups not differ during reward delivery? First, in a well-learned task like ours reward delivery generates little BOLD response in reward circuits [84], and fMRI may be insufficiently sensitive to detect possible group differences there. Second, among adults with impulsive and aggressive traits rewards generate enhanced BOLD responses in dopaminergic reward circuits [32]. Our patients had impulsive and aggressive traits (Table 1), predicting *enhanced* reward-circuit function [32], and they also were addicted, predicting *diminished* reward-circuit responses [33], [18]. Perhaps reflecting both opposing influences, our patients' reward-related neural activity in NAc did not differ from controls'.

Complicating the concept of reward insensitivity among patients is that they and controls rated themselves similarly “happy” upon winning in the CBG (Fig. 2D). Although the VAS was sufficiently sensitive to detect different emotional responses to win-vs.-loss, it may not have been sensitive enough to detect different emotional responses of patients and controls to wins. It also may be that “reward insensitivity” in human beings is not a short-term emotional response to a series of individual rewarding events, but a cumulative failure of such events to raise one's overall mood from sustained dysphoria. Future research may clarify this question.

#### Experiencing Losses

As the two groups experienced losses, patients' BOLD responses to loss exceeded controls'. Controls activated no regions more than patients; meanwhile, patients activated right DLPFC and left OFC, as well as brainstem, cerebellum, and temporal and parietal structures, more than controls (Table 11). Koob and Volkow [33] propose that addiction activates a CRF-dependent brain stress system and imposes reward insensitivity. Our findings extend those predictions, indicating that ASD youths also develop loss hypersensitivity, perhaps facilitated by a hyperactive stress system, and further contributing to subjective dysphoria.

#### Another View

Despite its great value, functional imaging provides an incomplete assessment of neural function [86]. A different interpretation of our data could be that patients have a fast, automated reward response that recruits few cognitive-control resources and allows unconstrained pursuit of rewards, a “reward *hyper*sensitivity”. Conversely, controls could have a fast, effective, automated response to punishment, while patients, generating a less effective response, need to call upon other processing resources. Our data cannot rule out this explanation, but the data better fit the Koob- Volkow [33] formulation. Built on extensive animal and human studies of addiction, that formulation accounts for drug-impaired prefrontal cognitive functions, drug-induced activation of a CRF stress system, drug-altered stimulation thresholds in reward circuits (reward insensitivity), and subjective dysphoria.

### Similar Risk-Taking among Patients and Controls

Although our patients were in treatment for unconstrained real-life risk-taking, substantiated with measures of pathological aggressiveness, impulsiveness, and substance, legal, and conduct problems (Table 1), and although in laboratory tasks such youths take more risks than controls [14], [15], patients and controls did not differ in risk-taking during CBG. In a similar task adult psychopaths made more risky responses than non-psychopaths, but that difference was eliminated when the experimenters required 5 seconds of deliberation before responses [87]. CBG's required 4-sec pre-response deliberation similarly may have reduced patients' excessive risk-taking. CBG's immediate rewards and punishments, punishments larger than rewards, and escalating frequency of punishments also may have limited risk-taking. However, despite behaving similarly, while processing risky decisions and their consequences, patients and controls clearly deployed different neural resources. Moreover, that behavioral similarity meant that the groups experienced similar numbers of wins, and of losses; that experimental advantage assured that neither group had greater frustration and different brain activity because of more losses.

### Limitations

Various concerns and criticisms may limit conclusions from our data. First, a patient-control socioeconomic status (SES) difference (Table 1) might have made our monetary rewards more important to patients than to controls. However, although the “happy-sad” self-ratings for wins differed significantly from those for losses (Fig. 2D), happy-sad ratings did not differ by group, suggesting that group SES differences did not strongly influence them.

Second, higher attention-deficit scores among patients might suggest that their neural hypoactivity during decision-making and winning reflected mere inattention. We think this unlikely. First, patients' mean reaction time (Table 2) did not differ from controls'. Also, although patients were significantly less likely to respond in the required time, the actual difference was very small (mean 1.1 responses in 90 trials; p = 0.049).

Third, our design could not dissect apart the roles of drugs and non-pharmacologic (*e.g.*, genetic) influences on our findings. Had we collected “pure” samples of youths with CD but not SUD, and SUD but not CD, we might have appeared to address that issue. However, the very strong comorbidity of CD and SUD means that such groups would be comprised of quite atypical cases, from whom findings could not generalize widely. Meanwhile, our patients resembled those who commonly present in clinical settings, to whom our findings do generalize. However, future studies should consider the probable heterogeneity in patients like ours.

Fourth, patients had widespread dysfunction in many brain structures. We cannot identify one, or a few, structures responsible for ASD.

Fifth, considered alone, patients' weaker neural responses during decision-making, or during wins, might have reflected some general inability to generate BOLD responses. However, patients had *stronger* BOLD responses to loss than controls (Table 11). Apparently, depending on win-or-loss stimulus conditions, patients were able to generate strong hemodynamic responses.

Sixth, did our AlphaSim [51] thresholds adequately minimize false-positive results? In the decision period, the win period, and the loss period we considered both control>patient, and patient>control, contrasts. For each period some 1000 to 6000 voxels exceeded the AlphaSim threshold in one contrast (Tables 5, 8, 11), while zero voxels did so in the other. These repeated findings of zero activation strongly suggest a low prevalence of false positives, while repeated findings of zero-vs.-considerable activation in the paired comparisons do suggest real group differences. Also, our AlphaSim-generated cluster threshold was similar to others recently published [88]; [ also see 52]–[57]. AlphaSim findings apparently have validity.

Seventh, jittered fixation screens between the green-light response and the red-light win-or-loss periods (Fig. 1B) might better have deconvolved hemodynamic curves, minimizing the influence of prior events on win-or-loss images. However, we avoided such jittering because it would variably delay reinforcements and punishments, unpredictably affecting response learning. Fortunately, all wins, and all losses, were preceded by an identical decision to make a right-hand response, and by its execution, assuring a similar hemodynamic carry-over for wins and losses. This similar carry-over could not explain why, for example, wins produced weaker, and losses produced stronger, neural responses in patients, compared to controls.

Eighth, our decision analyses used all 90 decision trials. Because our win and loss analyses each examined only about 25 trials (Fig. 2B), their lower power strongly calls for replication.

Finally, of course, data from boys cannot be generalized to girls.

### Clinical Implications

Extensive research suggests that adolescent ASD is a genetically-initiated, drug-exacerbated, persisting disposition to make risky antisocial and substance-use decisions. Our findings suggest that abnormal neural processing of risky decisions, rewards, and losses may contribute to these patients' frequent, dangerous relapses [89]. Such patients can improve during, and for some months after, treatment [90], but the brain abnormalities reported here may persist into adulthood [21], leaving these patients continually vulnerable to substance and antisocial relapse.

## References

[1] Drug and Alcohol Services Information System TEDS: (Treatment Episode Data Set).. http://www.oas.samhsa.gov/DASIS.htm.

[2] Disney ER, Elkins IJ, McGue M, Iacono WG (1999). Effects of ADHD, conduct disorder, and gender on substance use and abuse in adolescence.. Am J Psychiatry.

[3] Costello EJ, Mustillo S, Erkanli A, Keeler G, Angold A (2003). Prevalence and development of psychiatric disorders in childhood and adolescence.. Arch Gen Psychiatry.

[4] Crowley TJ, Gelhorn H, Koob G, Le Moal M, Thompson R (2010). Antisocial drug dependence.. in chief, *Encyclopedia of Behavioral Neuroscience.*.

[5] Cadoret RJ, Yates WR, Troughton E, Woodworth G, Stewart MA (1996). An adoption study of drug abuse/dependency in females.. Compr Psychiatry.

[6] Slutske WS, Heath AC, Dinwiddies SH, Madden PAF, Bucholz KK (1998). Common genetic risk factors for conduct disorder and alcohol dependence.. J Abnorm Psychol.

[7] Hicks BM, Krueger RF, Iacono WG, McGue M, Patrick CJ (2004). Family transmission and heritability of externalizing disorders: A twin-family study.. Arch Gen Psychiatry.

[8] Button TMM, Rhee SH, Hewitt JK, Young SE, Corley RP (2007). The role of conduct disorder in explaining the comorbidity between alcohol and illicit drug dependence in adolescence.. Drug Alcohol Depend.

[9] Bamford NS, Zhang H, Joyce JA, Scarlis CA, Hanan W (2008). Repeated exposure to methamphetamine causes long-lasting presynaptic corticostriatal depression that is renormalized with drug readministration.. Neuron.

[10] Hansen HH, Krutz B, Sifringer M, Stefovska V, Bittigau P (2008). Cannabinoids enhance susceptibility of immature brain to ethanol neurotoxicity.. Ann Neurol.

[11] Sullivan EV, Pfefferbaum A (2005). Neurocircuitry in alcoholism: A substrate of disruption and repair.. Psychopharmacology.

[12] Volkow ND, Fowler JS, Wang GJ, Baler R, Telang F (2009). Imaging dopamine's role in drug abuse and addiction.. Neuropharmacology.

[13] Larm P, Hodgins S, Larsson A, Samuelson YM, Tengstrom A (2008). Long-term outcomes of adolescents treated for substance misuse.. Drug Alcohol Depend.

[14] Lane SD, Cherek DR (2001). Risk taking by adolescents with maladaptive behavior histories.. Exp Clin Psychopharmacol.

[15] Crowley TJ, Raymond KE, Mikulich-Gilbertson SK, Thompson LL, Lejuez CW (2006). A risk-taking “set” in a novel task among adolescents with serious conduct and substance problems.. J Am Acad Child Adolesc Psychiatry.

[16] American Psychiatric Association (2000). Diagnostic and Statistical Manual of Mental Disorders 4^th^ Edition, Text Revision.

[17] Galvan A, Hare T, Voss H, Glover G, Casey BJ (2007). Risk-taking and the adolescent brain: Who is at risk?. Dev Sci.

[18] Kringelbach ML (2005). The human orbitofrontal cortex: Linking reward to hedonic experience.. Neuroscience.

[19] Finger EC, Marsh AA, Mitchell DG, Reid ME, Sims C (2008). Abnormal ventromedial prefrontal cortex function in children with psychopathic traits during reversal learning.. Arch Gen Psychiatry.

[20] Elliott R, Deakin B (2005). Role of the orbitofrontal cortex in inhibitory processing and inhibitory control: Evidence from functional magnetic resonance imaging studies in healthy human subjects.. Internat Rev of Neurobiology.

[21] Bjork JM, Momenan R, Smith AR, Hommer DW (2008). Reduced posterior mesofrontal cortex activation by risky rewards in substance-dependent patients.. Drug Alcohol Depend.

[22] Eshel N, Nelson EE, Blair RJ, Pine DS, Ernst M (2007). Neural substrates of choice selection in adults and adolescents: Development of the ventrolateral prefrontal and anterior cingulate cortices.. Neuropsychologia.

[23] Blair RJR (2008). The amygdala and ventromedial prefrontal cortex: Functional contributions to dysfunction in psychopathy.. Phil Trans R Soc B.

[24] Banich MT, Crowley TJ, Thompson LL, Jacobson BL, Liu X (2007). Brain activation during the Stroop task in adolescents with severe substance and conduct problems: A pilot study.. Drug Alcohol Depend.

[25] Tapert SF, Schweinsburg AD, Drummond SP, Paulus MP, Brown SA (2007). Functional MRI of inhibitory processing in abstinent adolescent marijuana users.. Psychopharmacology.

[26] McNamee RL, Dunfee KL, Luna B, Clark DB, Eddy WF (2008). Brain activation, response inhibition, and increased risk for substance use disorder.. Alcohol Clin Exp Res.

[27] Sterzer P, Stadler C, Poustka F, Kleinschmidt A (2007). A structural neural deficit in adolescents with conduct disorder and its association with lack of empathy.. NeuroImage.

[28] Huebner T, Vloet TD, Marx I, Konrad K, Fink GR (2008). Morphometric brain abnormalities in boys with conduct disorder.. J Am Acad Child Adolesc Psychiatry.

[29] Benegal V, Antony G, Venkatasubramanian G, Jayakumar PN (2006). Gray matter volume abnormalities and externalizing symptoms in subjects at high risk for alcohol dependence.. Addiction Biology.

[30] Boes AD, Bechara A, Tranel D, Anderson SW, Richman L (2009). Right ventromedial prefrontal cortex: A neuroanatomical correlate of impulse control in boys.. Soc Cogn Affect Neurosci.

[31] Boes AD, Tranel D, Anderson SW, Nopoulos P (2008). Right anterior cingulate: A neuroanatomical correlate of aggression and defiance in boys.. Behav Neurosci.

[32] Buckholtz J, Treadway MT, Cowan RL, Woodward ND, Benning SD (2010). Mesolimbic dopamine reward system hypersensitivity in individuals with psychopathic traits.. Nature Neuroscience.

[33] Koob GF, Volkow ND (2010). Neurocircuitry of addiction.. Neuropsychopharmacology.

[34] Crowley TJ (1972). The reinforcers for drug abuse: Why people take drugs.. Comprehensive Psychiatry.

[35] Du YP, Dalwani M, Wylie K, Claus E, Tregellas JR (2007). Reducing susceptibility artifacts in fMRI using volume-selective z-shim compensation.. Magn Reson Med.

[36] Achenbach TM, Howell CT, Quay HC, Conners CK (1991). National survey of problems and competencies among four- to sixteen-year-olds: Parents' reports for normative and clinical samples.. Monogr Soc Res Child Dev.

[37] Crowley TJ, Mikulich SK, Ehlers KM, Whitmore EA, Macdonald MJ (2001). Validity of structured clinical evaluations in adolescents with conduct and substance problems.. J Am Acad Child Adolesc Psychiatry.

[38] Shaffer D, Fisher P, Lucas CP, Dulcan MK, Schwab-Stone ME (2000). NIMH Diagnostic Interview Schedule for Children Version IV (NIMH DISC-IV): Description, differences from previous versions, and reliability of some common diagnoses.. J Am Acad Child Adolesc Psychiatry.

[39] Robins LN, Wing J, Witchen HU, Helzer JE, Babor TF (1988). The Composite International Diagnostic Interview: An epidemiologic instrument suitable for use in conjunction with different diagnostic systems and in different cultures.. Arch Gen Psychiatry.

[40] Cottler LB, Schuckit MA, Helzer JE, Crowley TJ, Woody G (2003). The DSM-IV field trial for substance use disorders: Major results.. Drug Alcohol Depend.

[41] Compton WM, Cottler LB, Dorsey KB, Spitznagel EL, Mager DE (1996). Comparing assessments of DSM-IV substance dependence disorders using CIDI-SAM and SCAN.. Drug Alcohol Depend.

[42] Alessi ME, McManus M, Grapentine WL, Brickman A (1984). The characterization of depressive disorders in serious juvenile offenders.. J Affective Disord.

[43] Carroll BJ, Feinberg M, Smouse PE, Rawson SG, Greden JF (1981). The Carroll rating scale for depression, I: Development, reliability and validation.. Br J Psychiatry.

[44] Hollingshead AB, Redlich FC (1958). Social class and mental illness: A community study.

[45] Wechsler D (1999). Wechsler Abbreviated Scale of Intelligence.

[46] Eysenck SBG, Eysenck HJ (1980). Impulsiveness and venturesomeness in children.. Personality and Individual Differences.

[47] Reitan RM, Wolfson D (1985). The Halstead-Reitan neuropsychological test battery.

[48] Dale AM (1999). Optimal experimental design for event-related fMRI.. Hum Brain Mapp.

[49] Ashburner J, Flandin G, Henson R, Kieble S, Kilner J SPM5 Manual. Wellcome Trust Center for Neuroimaging.. http://www.fil.ion.ucl.ac.uk/spm/.

[50] Raine A, Yang Y (2006). Neural foundations to moral reasoning and antisocial behavior.. Soc Cogn Affect Neurosci.

[51] Ward BD (June 2000). Simultaneous inference for fMRI data.. http://afni.nimh.nih.gov/pub/dist/doc/manual/AlphaSim.pdf.

[52] Cristiana C-P, Melvyn AG, Jody CC (2007). FMRI Reveals a dissociation between grasping and perceiving the size of real 3D objects.. PLoS ONE.

[53] Yan C, Liu D, He Y, Zou Q, Zhu C (2009). Spontaneous brain activity in the default mode network is sensitive to different resting-state conditions with limited cognitive load.. PLoS ONE.

[54] Morgan VL, Mishra A, Newton AT, Gore JC, Ding Z (2009). Integrating Functional and Diffusion Magnetic Resonance Imaging for analysis of structure-function relationship in the human language network.. PLoS ONE.

[55] Depue BE, Curran T, Banich MT (2007). Prefrontal regions orchestrate suppression of emotional memories via a two-phase process.. Science.

[56] Poldrack RA, Fletcher PC, Henson RN, Worsley KJ, Brett M (2008). Guidelines for reporting an fMRI study.. NeuroImage.

[57] Wang K, Liang M, Wang L, Tian L, Zhang X (2007). Altered functional connectivity in early Alzheimer's Disease: A resting-state fMRI study.. Human Brain Mapp.

[58] NRW Research Group for Hemispheric Specialization (2003). Münster T2T-Converter.. http://wwwneuro03.uni-muenster.de/ger/t2tconv.

[59] Petrides M (2005). Lateral prefrontal cortex: Architectonic and functional organization.. Philos Trans R Soc Lond B Biol Sci.

[60] Ridderinkhof KR, Ullsperger M, Crone EA, Nieuwenhuis S (2004). The role of the medial frontal cortex in cognitive control.. Science.

[61] Ranson SW, Clark SL (1959). The anatomy of the nervous system: Its development and function, 10^th^ Ed.

[62] Neto LL, Oliveira E, Correia F, Ferrira AG (2008). The human nucleus accumbens: Where is it? A stereotatic, anatomical and magnetic resonance imaging study.. Neuromodulation.

[63] Mai JK, Paxinos G, Voss T (2008). Atlas of the Human Brain.

[64] Childress AR, Miller W, Carroll K (2006). What can human brain imaging tells us about vulnerability to addiction and to relapse?. Rethinking substance abuse: What the science shows and what we should do about it.

[65] Augustine JR (1996). Circuitry and functional aspects of the insular lobe in primates including humans.. Brain Res Brain Res Rev.

[66] Craig AD (2009). How do you feel – now? The anterior insula and human awareness.. Nature Reviews Neuroscience.

[67] Paulus MP, Rogalsky C, Simmons A, Feinstein JS, Stein MB (2003). Increased activation in the right insula during risk-taking decision making is related to harm avoidance and neuroticism.. NeuroImage.

[68] Sanfey AG (2007). Social decision-making: Insights from game theory and neuroscience.. Science.

[69] Moll J, Krueger F, Zahn R, Pardini M, de Oliveira-Souza R (2006). Human fronto-mesolimbic networks guide decisions about charitable donation.. Proc Natl Acad Sci U S A.

[70] Ernst M, Nelson EE, McClure EB, Monk CS, Munson S (2004). Choice selection and reward anticipation: An fMRI study.. Neuropsychologia.

[71] Matthews SC, Simmons AN, Lane SD, Paulus MP (2004). Selective activation of the nucleus accumbens during risk-taking decision making.. NeuroReport.

[72] Preuschoff K, Quartz SR, Bossaerts P (2008). Human insula activation reflects risk prediction errors as well as risk.. J Neurosci.

[73] Belin D, Jonkman S, Dickinson A, Robbins TW, Everitt BJ (2009). Parallel and interactive learning processes within the basal ganglia: Relevance for the understanding of addiction.. Behav Brain Res.

[74] Krain AL, Wilson AM, Arbuckle R, Castellanos FX, Milham MP (2006). Distinct neural mechanisms of risk and ambiguity: A meta-analysis of decision-making.. NeuroImage.

[75] Robbins TW, Ershe KD, Everitt BJ (2008). Drug addiction and the memory systems of the brain.. Ann N Y Acad Sci.

[76] Savage LM, Ramos RL (2009). Reward expectation alters learning and memory: The impact of the amygdala on appetitive-driven behaviors.. Behav Brain Res.

[77] Nachev P, Kennar C, Husain M (2008). Functional role of the supplementary and pre-supplementary motor areas.. Nature Reviews Neuroscience.

[78] Parvizi J, Van Hoesen GW, Buckwalter J, Damasio A (2006). Neural connections of the posteromedial cortex in the macaque.. Proc Natl Acad Sci U S A.

[79] Cavanna AE, Trimble MR (2006). The precuneus: A review of its functional anatomy and behavioural correlates.. Brain.

[80] Baillieux H, De Smet HJ, Paquier PE, De Deyn PP, Marien P (2008). Cerebellar neurocognition: Insights into the bottom of the brain.. Clin Neurol Neurosurg.

[81] Koeneke S, Pedroni AF, Dieckmann A, Bosch V, Jäncke L (2008). Individual preferences modulate incentive values: Evidence from functional MRI.. Behav Brain Funct.

[82] Killgore WD, Yurgelun-Todd DA (2005). Developmental changes in the functional brain responses of adolescents to images of high and low-calorie foods.. Dev Psychobiol.

[83] David SP, Munafò MR, Johansen-Berg H, Smith SM, Rogers RD (2005). Ventral striatum/nucleus accumbens activation to smoking-related pictorial cues in smokers and nonsmokers: a functional magnetic resonance imaging study.. Biol Psychiatry.

[84] Galvan A, Hare TA, Davidson M, Spicer J, Glover G (2005). The role of ventral frontostriatal circuitry in reward-based learning in humans.. J Neurosci.

[85] Balleine BW, Delgado MR, Hikosaka O (2007). The role of dorsal striatum in reward and decision-making.. J Neurosci.

[86] Lauritzen M, Gold L (2003). Brain function and neurophysiological correlates of signals used in functional neuroimaging.. J Neurosci.

[87] Newman JP, Patterson M, Kosson D (1987). Response perseveration in psychopaths.. J Abnorm Psychol.

[88] Bennett CM, Wolford GL, Miller MB (2009). The principled control of false positives in neuroimaging.. Soc Cogn Affective Neurosci.

[89] Tomlinson KL, Brown SA, Abrantes A (2004). Psychiatric comorbidity and substance use treatment outcomes of adolescents.. Psychology of Addictive Behaviors.

[90] Crowley TJ, Mikulich SK, Macdonald M, Young SE, Zerbe GO (1998). Substance-dependent, conduct-disordered adolescent males: Severity of diagnosis predicts two-year outcome.. Drug Alcohol Depend.

